# The Knowledge and Attitude of Patients Diagnosed with Epithelial Ovarian Cancer towards Genetic Testing

**DOI:** 10.3390/ijerph18052312

**Published:** 2021-02-26

**Authors:** Wonkyo Shin, Gowoon Jeong, Yedong Son, Sang-Soo Seo, Sokbom Kang, Sang-Yoon Park, Myong Cheol Lim

**Affiliations:** 1 Center for Gynecologic Cancer, National Cancer Center, 323 Ilsan-ro, Goyang 10408, Korea; 12958@ncc.re.kr (W.S.); 11821@ncc.re.kr (G.J.); ssseomd@ncc.re.kr (S.-S.S.); sokbom@ncc.re.kr (S.K.); parksang@ncc.re.kr (S.-Y.P.); 2 College of Nursing, Woosuk University, Wanju 55338, Korea; cokitose@naver.com; 3 Division of Precision Medicine, Research Institute, National Cancer Center, 323 Ilsan-ro, Goyang 10408, Korea; 4 Department of Cancer Control & Population Health, Graduate School of Cancer Science and Policy, National Cancer Center, Goyang 10408, Korea; 5 Center for Clinical Trials, Hospital, National Cancer Center, 323 Ilsan-ro, Goyang 10408, Korea; 6 Division of Tumor Immunology, National Cancer Center, 323 Ilsan-ro, Goyang 10408, Korea

**Keywords:** ovarian cancer, next generation sequencing, knowledge, attitude, genetic test

## Abstract

This study assessed the knowledge and attitude of patients with ovarian cancer (OC) toward OC and next generation sequencing (NGS). The data, including characteristics of patients, their knowledge about OC and their knowledge and attitude of NGS, were collected from June to October 2018. Of the 103 participants, 70.9% (*n* = 73) had cancer within the second-degree relatives, and 18.4% (*n* = 19) had *BRCA* pathogenic mutations. The percentage of right answer for the knowledge about OC and NGS was 64.7% (11/17) and 50% (6/12), respectively. The median number of patients who had positive expectations for the genetic test was 34 (range, 22–44). Based on a first-degree familial history, patients had a different degree of knowledge about OC (11 vs. 8.5, *p* = 0.026) and NGS (6.5 vs. 5, *p* = 0.011), but patients with a *BRCA* pathogenic mutation did not have a different degree of knowledge about OC and NGS panel testing. High-income families had a more positive attitude towards the genetic test than low-income families (*p* = 0.005). Women with OC do not have enough knowledge about OC (11/17, 64.7%) and NGS (6/12, 50%) but they showed a positive attitude toward the NGS test. These women need OC and NGS educational intervention.

## 1. Introduction

Genetic testing is rapidly and widely applied in cancer diagnosis and treatment [1,2]. Knowledge of accumulated genetics reveals the biological mechanisms of cancer and how to apply diagnosis and treatment. About fifteen to twenty percent of epithelial ovarian cancer (OC) patients have hereditary diseases, and ongoing research has identified more genes associated with OC [3]. The standard treatment for ovarian cancer is primary cytoreductive surgery followed by platinum-based(PCS) and adjuvant chemotherapy. A few years ago, neoadjuvant chemotherapy (NAC) before surgery was also introduced as a standard treatment. NAC is effective in reducing the tumor burden before surgery, so it can induce the reduction of complications related to surgery in patients, and there is no significant difference in the overall prognosis between NAC and PCS [4].

Since a poly (ADP-ribose) polymerase inhibitor has recently achieved remarkable results in *BRCA*-mutated OC patients’ therapy [5,6], the genes associated with OC have been implicated in OC screening and treatment [7]. Patients with OC need genetic testing to develop their treatment plan, and they need screening as part of their familial risk evaluation. According to National Comprehensive Cancer Network (NCCN) guidelines, since 2017 next generation sequencing (NGS) has been recommended for testing for mutations and screening for familial risk in patients diagnosed with epithelial OC [8].

According to recently reported data, however, only fifteen to twenty percent of patients who require genetic testing are getting tested [9]. Subsequent studies have endeavored to determine whether patients who receive genetic counseling are more likely to undergo genetic testing [10,11]. According to a patient survey, genetic counseling prior to genetic testing did not affect the patients’ attitudes toward genetic testing. Other studies have measured knowledge about genetic testing in general populations, or patients with noncommunicable diseases. Researchers found little relation between individual disease status and genetic testing knowledge [12,13]. Breast cancer patients with a high risk were more satisfied with interdisciplinary genetic outpatient consultations than breast cancer patients with a low risk [14]. This study investigates OC patients’ knowledge and attitude towards NGS.

## 2. Materials and Methods

### 2.1. Participants and Procedure

This study had a cross-sectional design. We created a questionnaire, every item of which was validated by four gynecologists. Before conducting a full-scale survey we tested the difficulty of the questionnaire on three patients and adjusted the words used. This study was approved by the institutional review board at the National Cancer Center of Korea (NCC2018-0090). Patients who were newly diagnosed with epithelial ovarian, fallopian or primary peritoneal cancer were the candidates for this survey. When a patient first visited an outpatient clinic after discharge, participants were asked to complete a questionnaire, after giving written informed consent. To reduce the possibility of bias between surveys, a nurse with a genetic testing certificate conducted the questionnaire. From June to October 2018, a total of 103 patients agreed to participate in this study. The questionnaire included a total of 38 items: 17 about the OC; 15 about the NGS; and nine about attitudes towards NGS. However, three items about the NGS were difficult to quantify and, therefore, were excluded from the analysis. Participants who were newly diagnosed with OC and were undergoing genetic testing were included in this study, but patients with metastatic OC were excluded.

### 2.2. Measures

Questions about OC included 17 items, 15 of which were designed to measure patient knowledge about NGS. Patients’ knowledge about OC and NGS was assessed using true/false items that had a “don’t know” option (which was coded as incorrect). In the NGS knowledge measurement category, three items were not included in the score; they measured patients’ experiences but not their level of knowledge. Cronbach’s alpha values were obtained to confirm the consistency of the items (OC knowledge items were 0.753, NGS items were 0.6446, attitude towards NGS items were 0.659). The attitudes section included a total of nine items, three of which confirmed positive attitudes towards genetic testing and six of which indicated negative attitudes. Each item was rated on a five-point Likert scale ranging from “absolutely no” (scored as 1) to “absolutely yes” (scored as 5). The questionnaire items about attitude were separated into positive and negative questions, and the responses to negative questions were coded reversely.

### 2.3. Analysis

R-project for statistical computing (version 3.5.2, R Foundation, Vienna, Austria) was used to analyze the data. Descriptive statistics were used to summarize the characteristics of patients, their knowledge about OC and NGS and their attitudes towards NGS. Descriptive statistics were used to determine frequency counts, percentages, means and standard deviations. The Wilcoxon rank sum test and Kruskal-Wallis test were used to confirm the significance of each item. Spearman’s correlation was used to analyze the correlation between patients’ knowledge about OC and NGS and patients’ attitude towards NGS.

## 3. Results

Table 1 presents the clinico-pathological characteristics of participants. The respondents’ mean age of diagnosis was 53.11 ± 11.5 years. Of the total number of patients, 48 patients (46.6%) had their family history in first-degree and 25 patients (24.3%) had family history in second-degree. Ninety-eight patients (95.2%) had undergone genetic testing using NGS and 19 patients (18.5%) had the *BRCA1* or *BRCA 2* mutation. Ten patients (9.7%) were diagnosed with another malignancy.

Table 2 shows the socio-demographic characteristics of patients. Thirty-six patients (35%) had a university degree and above, and 42 patients (40%) had occupations. Most of the patients (*n* = 86, 83.5%) discussed their disease status with their family. More than half of the patients (*n* = 54, 52.4%) got knowledge and information about the disease from the internet, and 89 patients (86%) decided to receive NGS at the recommendation of medical staff.

Table 3 shows the score of knowledge about the ovarian cancer, NGS and attitude. The median score of self-rated knowledge about OC was 11 (range, 3–16) and that of NGS was 34 (range, 22–44). The median attitude score was 34.

The differences of knowledge about the EOC and NGS and attitude by clinical factors are shown in Table 4. The patients with a first-degree family history had a relatively high score concerning knowledge about OC (11 vs. 8.5, *p* = 0.026) and NGS (6.5 vs. 5, *p* = 0.011). However, there was no significant difference in their attitudes towards genetic testing scores. The presence of cancer in patients’ second-degree family history did not affect their knowledge or attitudes. Unmarried women had higher scores than married women in knowledge about OC (13 vs. 10, *p* = 0.004) There was no significant difference between the attitudes towards genetic testing scores of married and unmarried women. High income groups had a high score (*p* = 0.005) in their attitude towards NGS, but there was no difference in their knowledge between about OC and about NGS. Other socioeconomic and clinical factors did not show differences. Other factors not associated with knowledge and attitude included histology, disease stage, surgery and genetic mutation status.

**Figure 1:**
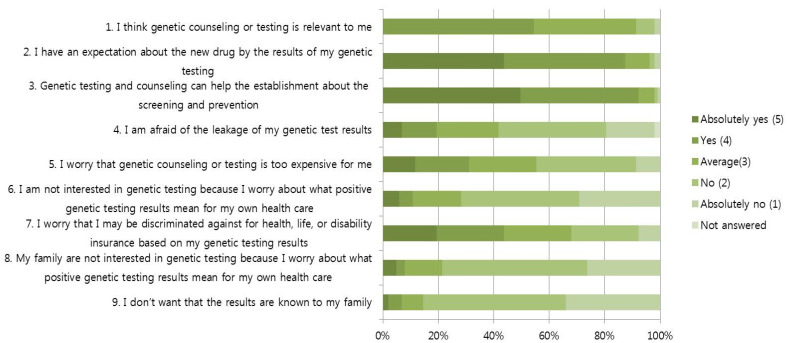
Attitude to next-generation sequencing.

Figure 1 presents the patients’ attitudes towards NGS. Patients responded positively to the positive effects of disease prevention and the opportunity to receive new treatments based on genetic testing. Of the total patients, 90 (87.4%) agreed or strongly agreed that patients should have the opportunity to receive new treatments based on genetic testing, and 95 patients agreed or strongly agreed that genetic testing could help screen for and detect other cancers early. Concerns about the negative effects of genetic testing tended to be low: 20 participants (19.4%) worried that their personal information would be leaked, 11 participants (10.2%) worried about negative test results, eight participants worried about the negative test results and 45 (43.7%) worried about economic disadvantages, such as the cost of insurance.

**Figure 2:**
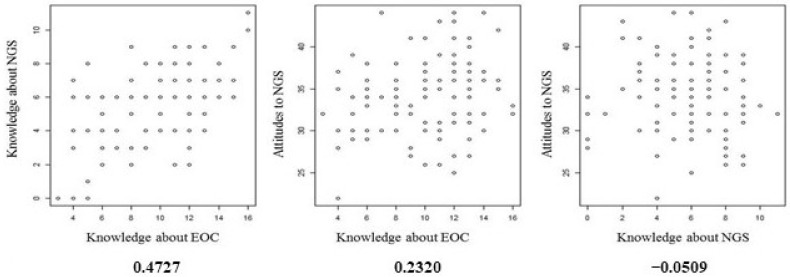
Attitude to next-generation sequencing.

Figure 2 shows the correlations between patients’ knowledge of OC and NGS and their attitudes towards NGS. There is some correlation between patients’ OC knowledge and their NGS knowledge, but there is no correlation between their attitude and knowledge scores.

## 4. Discussion

In the current study, cancer patients’ knowledge and attitudes towards genetic testing tended to have lower scores considered that the questions had an appropriate level of difficulty. Meanwhile, patients generally responded positively to new techniques or tests [10,11,12].

The standard treatment for ovarian cancer is PCS followed by adjuvant chemotherapy, or NAC followed by interval cytoreductive surgery. On the other hand, in recent years, as genetic knowledge about ovarian cancer has rapidly increased, poly (ADP-ribose) polymerase (PARP) inhibitors were introduced to treat ovarian cancer, and promising results were shown in patients with breast cancer gene 1 and 2 (*BRCA1/2*) mutation carriers [6,15]. Therefore, genetic testing of OC patients is an essential test for patients to receive appropriate treatment. In addition, their families also need to undergo genetic testing for cancer screening and prevention [16]. Cancer patients want to receive a variety of care and information about various treatments from their medical team, but doctors tend to underestimate patients’ needs. In the past, medical decisions were made based on a one-way flow of information from medical personnel to patients. However, in recent years, patients have been able to obtain information from various sources in various ways. Patients generally obtain medical information from medical staff first, and then from the internet or the media. In Korea, however, most patients obtain medical information from the internet [17,18]. There is not enough time for medical staff to discuss the patients’ disease treatment and care with them [19]. Our research showed that many patients and their families searched for information about their disease. Over 70% of patients got information about their disease through the internet or the media.

According to the results of this questionnaire research, patients’ decisions to undergo genetic testing were largely a result of healthcare providers’ recommendations but patients appeared to have inaccurate information or lacked the necessary communication with their medical staff to make informed decisions. When patients are diagnosed and treated, the medical staff must provide them with enough knowledge and information about genetic testing to help them make informed decisions about their care. Healthcare providers need to update their genetic knowledge about, and interpretation of, genetic test results to keep up with change [20]. Therefore, an educational program is needed to improve genetic-related knowledge for healthcare providers and patients. It will also be necessary for medical staff to spend enough time communicating more about knowledge and information with patients.

Research has been conducted on the categorization of patients’ searches for information about their diseases. The pattern of these information searches can be divided into five groups: intense, complementary, fortuitous, minimal and active avoiders [21,22]. Middle aged, highly educated female patients more actively searched for medical information. However, there were no statistical differences between patients’ level of information seeking activity based on their educational status, occupation or income in the fortuitous, minimal and active avoiders’ groups [23]. In this study, unmarried, female patients with a family history of cancer were more knowledgeable. This might be because they are more active information seekers, and it is necessary to make knowledge available to them.

Patients with a diagnosis of cancer in the family had relatively high knowledge about OC and genetic testing. Patients who had family members with breast cancer had a relatively high level of knowledge and a tendency to more actively undergo screening [24,25]. It is likely that information about the disease was shared within the family during the treatment of the previously diagnosed family member. It is important for patients’ family members to share health information so that they can identify the genetic risks of diseases like cancer. Encouraging patients to communicate about their health information with their family members would provide disease prevention opportunities within the family [26].

Unmarried women had higher knowledge scores for both OC and NGS. However, there was no difference in their attitude towards NGS. Although not examined in this analysis, unmarried women were relatively young, and it may have been easier for them to access information about OC and NGS than it was for married women, who were typically older [27].

In patients with high household incomes, attitudes toward genetic testing were positive. For low-income families, the cost of the test and treatment seemed to be a burden. In Korea, the cost of NGS testing for 25 gene mutations is 802,880 KRW. Because the patient bears 50% of the Korean national health insurance, she would be required to spend 401,440 KRW at the time of the examination. Generally, when patients are diagnosed with a serious disease, such as cancer, the Korean national health insurance system is designed so that patients pay only 5% of the total medical expenses, but this does not include the cost of NGS testing. If the same benefits were provided for NGS testing, patients would be able to access the screening without the burden of cost. This would allow for further screening and new treatment opportunities for patients and their families.

This study measured attitudes towards NGS: the positive attitudes about the positive effects of NGS and the low concern about negative effects. The result of this study is similar to those of a previous study [12,28]. The prior study showed that a higher knowledge level was associated with a positive attitude towards genetic tests [29]. Our results showed that there was a relative correlation between knowledge about OC and NGS, but there was no correlation between knowledge level and attitude towards NGS. As previously mentioned, patients receive a lot of inaccurate knowledge about disease and genetic testing from the internet, but the medical staff unilaterally make the medical decisions, and patients may only be interested in the positive aspects of genetic tests.

In the future, research should consider the diagnosis and treatment of diseases at the gene level and their actual clinical applications, and medical professionals should be obliged to convey the most recent and accurate knowledge to their patients. Recently, the PARP inhibitor has been the only treatment for *BRCA*-related genetic mutations, but as the NGS test becomes more common and information on various genes is accumulated, new treatment strategies are emerging. In addition, research and treatment related to genes according to metabolic pathway abnormalities are gradually increasing [30]. Based on the results of this study, we can produce educational materials about disease and self-care that can help patients.

The limitations of this study include the lack of information on patients who did not respond to the questionnaire and the lack of comparison between those who responded to the questionnaire and those who did not.

This questionnaire surveyed patients in the initial stage of diagnosis. In the future, if the same questionnaire is used after a diagnosis of recurrence in the same patients, we will be able to unambiguously identify the necessary aspects of the disease treatment process.

This is the first study of OC patients undertaking NGS in Korea. Although the sample size was relatively small, the study is meaningful because it examined the knowledge level of patients and their attitudes toward testing. Patients generally had expectations for new treatments through tests, but they were less concerned about personal information security.

## 5. Conclusions

The current study showed that patients do not have sufficient knowledge about OC and genetic testing to decide their treatment plan or discuss their disease with medical professionals. In addition, the questionnaire showed that patients expected that genetic testing would help with the treatment of their illness.

When patients who are diagnosed OC, their knowledge about disease is generally poor, so medical staff should offer information to patients about the disease and treatment courses. The results of this study can serve as a basis for developing educational materials for patients who are diagnosed OC.

## Figures and Tables

**Table 1:** Clinico-pathological characteristics (*N* = 103).

Variables	Items	*N*(%) or Mean ± SD
Age at diagnosis (year)		53.11 ± 11.56
ECOG	0, 1	88 (85.4)
	2, 3, 4	9 (8.8)
	Unknown	6 (5.8)
Family history in 1st degree	Yes	48 (46.6)
	No	40 (38.8)
	Unknown	15 (14.6)
Family history in 2nd degree	Yes	25 (24.3)
	No	63 (61.2)
	Unknown	15 (14.5)
NGS (germline)	None	79 (76.7)
	*BRCA1* PV/*BRCA2* PV	19 (18.5)
	Not performed	5 (4.8)
Histologic type	High grade serous	68 (66.0)
	Mucinous/Clear/Endometrioid/Others	35 (34.0)
FIGO stage	1	11 (10.7)
	2	8 (7.8)
	3	54 (52.4)
	4	22 (21.3)
	Unknown	8 (7.8)
Operation optimality	Microscopic	47 (45.6)
	<1 cm	46 (44.7)
	Unknown	10 (9.7)
Other cancer	Yes	10 (9.7)
	No	93 (90.3)

Note. ECOG = Eastern Cooperative Oncology Group; NGS = next generation sequencing; FIGO = Federation of Gynecology and Obstetrics; PV = pathogenic variant.

**Table 2:** Socio-demographic characteristics (*N* = 103).

Variables	Items	*N* (%)
Religion	Yes	73 (70.9)
	No	30 (29.1)
Educational status	Under Middle school	23 (22.3)
	High school	44 (42.7)
	University and above	36 (35.0)
Occupation	Yes	42 (40.8)
	No	61 (59.2)
Marital status	Unmarried	11 (10.7)
	Married	81 (78.6)
	Divorced and etc.	11 (10.7)
Monthly household income	under 2 million KRW	28 (27.2)
	2~5 million KRW	60 (28.2)
	Over 5 million KRW	15 (14.6)
Who is the main person counseling about your health status	Family	86 (83.5)
	Friends	17 (16.5)
	Medical staff	13 (12.6)
	None	1 (1.0)
	Etc.	3 (2.9)
Main source of health information *	Internet	54 (52.4)
	Media (TV, Radio, Paper)	19 (18.5)
	Family	9 (8.7)
	Friends	9 (8.7)
	Patients community	12 (11.6)
	Medical staff	27 (26.2)
	Etc.	3 (2.9)
Who recommends the genetic test *	Self	10 (9.7)
	Family or friends	3 (2.9)
	Medical staff	89 (86.4)
	Unknown	2 (1.9)

* Multiple response was permitted.

**Table 3:** Knowledge about the ovarian cancer and NGS and attitude towards NGS.

Categories	Number of Items	Median (Min–Max)
Knowledge about the ovarian cancer	17	11 (3–16)
Knowledge about the NGS	12	6 (0–11)
Attitude towards NGS	9	34 (22–44)

**Table 4:** The differences of knowledge about the ovarian cancer and NGS and attitude towards NGS by participants’ characteristics.

Variables	Items	Knowledge about the EOC		Knowledge about the NGS		Attitude towards NGS	
		Median (Min–Max)	*p*-Value	Median (Min–Max)	*p*-Value	Median (Min–Max)	*p*-Value
ECOG	0, 1	10 (3–16)	0.076	6 (0–11)	0.094	34.5 (22–44)	0.349
	2, 3, 4	12 (8–15)		8 (4–9)		32 (29–40)	
	Unknown						
Family history in 1st degree	Yes	11 (4–15)	0.026	6.5 (0–9)	0.011	35 (22–44)	0.769
	No	8.5 (3–16)		5 (0–11)		35 (25–44)	
	Unknown						
Family history in 2nd degree	Yes	12 (5–16)	0.284	7 (0–11)	0.015	36 (27–44)	0.241
	No	10 (3–15)		6 (0–9)		35 (22–44)	
	Unknown						
NGS (germline)	None	11 (3–16)	0.811	6 (0–11)	0.479	35 (22–44)	0.304
	*BRCA1* PV*/BRCA2* PV	11 (4–15)		7 (0–9)		34 (25–42)	
Histologic type	High grade serous	10 (3–16)		6 (0–11)		35 (22–44)	
	Mucinous/Clear /Endometrioid/Others	12 (4–16)	0.069	6 (0–10)	0.733	33 (22–44)	0.402
FIGO stage	1	12 (4–15)	0.323	6 (0–8)	0.516	34 (27–42)	0.300
	2	12 (6–14)		5.5 (4–7)		34.5 (27–40)	
	3	10.5 (3–16)		6 (0–11)		35 (22–44)	
	4	8.5 (4–14)		5.5 (0–9)		33.5 (22–41)	
	Unknown	10 (3–15)		6 (0–9)		34 (22–44)	
Operation	Microscopic	12 (4–16)	0.067	7 (0–10)	0.332	34 (27–44)	0.793
optimality	<1 cm	9.5 (3–16)	□	6 (0–11)	□	35 (22–44)	□
Other cancer	Yes	12 (4–14)	0.724	6 (0–8)	0.377	34 (27–40)	0.492
	No	10 (3–16)		6 (0–11)		35 (22–44)	
Religion	Yes	11 (3–16)	0.683	6 (0–10)	0.573	34 (22–44)	0.419
	No	10 (4–16)		6 (0–11)		35 (27–44)	
Educational status	Under middle school	10 (4–16)	0.434	10 (3–15)	0.210	12 (4–16)	0.451
	High school	7 (1–11)		6 (0–9)		6 (0–10)	
	University and above	33 (22–42)		35 (26–41)		34.5 (22–44)	
Occupation	Yes	11 (4–16)	0.198	6 (0–10)	0.805	35 (22–44)	0.087
	No	10 (3–16)		6 (0–11)		34 (22–44)	
Marital status	Unmarried ^a^	13 (11–16)	0.005	7 (5–10)	0.060	33 (30–44)	0.807
	Married ^b^	10 (3–16)	a > b	6 (0–11)		35 (22–44)	
	Divorced and etc. ^c^	10 (4–14)		5 (1–8)		36 (26–41)	
Monthly household	under 2 million KRW ^a^	10.5 (4–16)	0.774	6 (0–11)	0.706	32 (25–42)	0.005
income	2~5 million KRW ^b^	10.5 (3–16)		6 (0–10)		35 (22–44)	a < b, c
	Over 5 million KRW ^c^	12 (5–14)	□	6 (3–9)	□	36 (27–43)	□

^a^, ^b^ and ^c^ were used as groups for comparison of post-hoc test.

## Data Availability

Not applicable.
