# Addendum: Shin, W.; et al. The Knowledge and Attitude of Patients Diagnosed with Epithelial Ovarian Cancer towards Genetic Testing. *Int. J. Environ. Res. Public Health* 2021, *18*, 2312

**DOI:** 10.3390/ijerph18084043

**Published:** 2021-04-12

**Authors:** Wonkyo Shin, Gowoon Jeong, Yedong Son, Sang-Soo Seo, Sokbom Kang, Sang-Yoon Park, Myong Cheol Lim

**Affiliations:** 1 Center for Gynecologic Cancer, National Cancer Center, 323 Ilsan-ro, Goyang 10408, Korea; 12958@ncc.re.kr (W.S.); 11821@ncc.re.kr (G.J.); ssseomd@ncc.re.kr (S.-S.S.); sokbom@ncc.re.kr (S.K.); parksang@ncc.re.kr (S.-Y.P.); 2 College of Nursing, Woosuk University, Wanju 55338, Korea; cokitose@naver.com; 3 Division of Precision Medicine, Research Institute, National Cancer Center, 323 Ilsan-ro, Goyang 10408, Korea; 4 Department of Cancer Control & Population Health, Graduate School of Cancer Science and Policy, National Cancer Center, Goyang 10408, Korea; 5 Center for Clinical Trials, Hospital, National Cancer Center, 323 Ilsan-ro, Goyang 10408, Korea; 6 Division of Tumor Immunology, National Cancer Center, 323 Ilsan-ro, Goyang 10408, Korea

The authors would like to add the following information to the “Funding” section of their paper published in the *International Journal of Environmental Research and Public Health* [1]:

This research was funded by the National Cancer Center Grant (NCC-1911274).
